# The spatiotemporal organization of episodic memory and its disruption in a neurodevelopmental disorder

**DOI:** 10.1038/s41598-019-53823-w

**Published:** 2019-12-05

**Authors:** Marilina Mastrogiuseppe, Natasha Bertelsen, Maria Francesca Bedeschi, Sang Ah Lee

**Affiliations:** 1 Center for Mind/Brain Sciences (CIMeC), University of Trento, Rovereto, Italy; 2 Department of Human Studies, University of Trieste, Trieste, Italy; 3 Center for Neuroscience and Cognitive Systems (CNCS), Italian Institute of Technology, Rovereto, TN Italy; 4 Medical Genetic Unit, Woman-Child-Newborn Department, Fondazione IRCCS Ca ‘Granda Ospedale Maggiore Policlinico, Milan, Italy; 5 Department of Bio and Brain Engineering, Korea Advanced Institute of Science and Technology, Daejeon, Korea

**Keywords:** Cognitive control, Human behaviour

## Abstract

Recent theories of episodic memory (EM) posit that the hippocampus provides a spatiotemporal framework necessary for representing events. If such theories hold true, then does the development of EM in children depend on the ability to first bind spatial and temporal information? And does this ability rely, at least in part, on normal hippocampal function? We investigated the development of EM in children 2–8 years of age (Study 1) and its impairment in Williams Syndrome, a genetic neurodevelopmental disorder characterized by visuospatial deficits and irregular hippocampal function, (Study 2) by implementing a nonverbal object-placement task that dissociates the *what*, *where*, and *when* components of EM. Consistent with the spatiotemporal-framework view of hippocampal EM, our results indicate that the binding of *where* and *when* in memory emerges earliest in development, around the age of 3, and is specifically impaired in WS. Space-time binding both preceded and was critical to full EM (*what + where + when*), and the successful association of objects to spatial locations seemed to mediate this developmental process.

## Introduction

Episodic memory (EM) contains various details of an event, such as the objects or people involved (“what”), the spatial setting (“where”) and the temporal sequence (“when”) in which the event unfolded. To create an EM representation, however, it is not enough to independently remember such details; they must be remembered as a coherently bounded, continuous episode. It is widely known that the hippocampal formation (HF) is crucial for memory binding processes and that hippocampal damage disrupts such processes^1–3^. At the same time, the HF has been intensively studied as the neural basis of spatial navigation in both humans and nonhuman animals^4–6^. To reconcile the role of the hippocampus in EM and spatial navigation, some researchers have suggested that the HF uses space and time as a primary scaffold for coding episodic memories and that other event-defining components are subsequently incorporated into this spatiotemporal framework^7,8^.

As animals move through their surroundings, representations of sequentially visited locations (and the properties that define those locations) contributes to a memory of traveled navigational paths. In the same way, episodic memories are created while individuals interact with their environment in specific contexts and settings. Although the HF has long been regarded as critical for spatial mapping (e.g.,^4,5^), there is now a large consensus that it is also involved in the temporal organization of memories. Recent findings illustrating that hippocampal and entorhinal cells represent episodic time^9–16^ and studies showing specific impairment in the temporal organization of memories in patients with hippocampal damage (e.g.,^17^), provide considerable support for the HF’s role in representing both spatial and temporal information.

Due to the physical nature of our experiences, humans and other animals cannot avoid being immersed in a spatiotemporal context at all times. Therefore, without the tight binding of spatial locations with time (and the objects that co-occur at each timepoint) to guide our goal-oriented behavior, we would end up wandering helplessly about, with no contiguity in the memory of our own experiences. If a spatiotemporal context represented by basic navigation mechanisms provides a scaffold even for coherent memories of non-navigational experiences, it follows that the ability to bind spatial and temporal information into a continuous representation is a requisite for EM. From a developmental perspective, therefore, it can be hypothesized that the ability to bind *where* and *when* should develop first, before the ability to represent a fully integrated memory of an episode (e.g., *what *+ *where* + *when*).

Previous studies on memory development in children have shown that while basic recognition abilities appear to be already present early in infancy, both for objects^18–21^ and spatial locations^22^, associative memory binding processes have a longer, more protracted maturation^18,23–27^. The variegated developmental trajectories of hippocampal subregions^28,29^ and the frontal-hippocampal network (e.g.,^30^) produce a gradual yet differentiated emergence of distinct memory processes and competences that, once integrated, can support a rich, coherent adult-like EM by around 9-to-11 years of age. However, studies with wider age ranges e.g.,^18^ suggest that memory for spatial contexts of events may occur earlier in development.

Olson and Newcombe^31^ suggested that among all the stimuli that are processed by our perceptual systems, spatial information might be more biologically salient and therefore play an organizing role in the structuring of our memories across development. Whether due to biological salience or an intrinsic feature of navigation-based hippocampal representations, there may be an advantage for binding space with time in early development. Such a process may provide the basis for episodic time, allowing memories of what happened (and the objects and people involved) to be remembered in a spatiotemporally continuous way. Moreover, if such abilities rely on the HF, subjects with atypical hippocampal development may be impaired in representing episodic time, particularly in the binding of spatial and temporal information.

**Figure 1:**
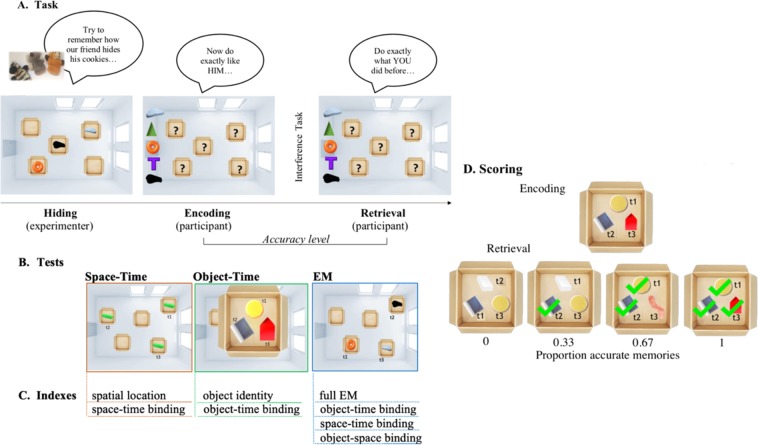
Episodic Memory Task. (**A)** The experimenter sequentially placed three objects in boxes laid out in the experimental room while the participant was watching (demonstration phase), and then the participant was asked to copy the experimenter by placing the objects themselves (encoding phase). After a delay (and verbal interference task), the participant was asked to re-enact his own previous actions (retrieval phase). (**B)** Each experimental session was composed of three tests: (i) The Space-Time Test required the combination of location and temporal sequence information but kept the object identity constant (i.e., by using identical objects); (ii) The Object-Time Test involved different objects but held the spatial location constant (i.e., by using just one box); (iii) The EM Test required the binding of information concerning object identity, spatial location, and temporal sequence into one representation. (**C)** We calculated the following indexes: the extent to which participants recognize the single components (object identity or spatial location), and their ability to bind them in a temporal sequence (space-time; object-time; EM). By separately analyzing bounded components in the EM Test it was possible to further investigate the effect of object-space, object-time and space-time binding on EM representation. (**D)** The indices were calculated by comparing the subject’s behavior between the encoding and retrieval phases (“proportion of accurate responses”). In the example above, the object-time binding index was calculated by assigning points for each correctly-remembered object in its correct temporal order (see Supplementary Information for a detailed description of the scoring).

The present study explored the above possibilities by investigating the developmental origins of EM in both typical and atypical development. In Study 1, we tested the binding of *what, where*, and *when* information in memory, across typically developing children between the ages 2 to 8, using a nonverbal object-hiding task (Figure1). In Study 2, we investigated how atypical hippocampal development may affect the development of episodic memory binding by conducting a similar test in young adult subjects with Williams Syndrome (WS), a genetic neurodevelopmental disorder characterized by a severe deficit in spatial cognition and irregular hippocampal function. If the HF is critical to the spatiotemporal binding of EM, it follows that WS subjects may be impaired in combining *where* + *when*, which consequently disrupts their EM. In contrast, WS may be relatively spared in their recognition memory, such as their ability to remember people and objects.

## Study 1: Typical Development of EM Binding Components

Previous studies have reported that episodic memory in typically-developing (TD) children begins to mature around 6 years of age and reaches adult-like levels at around 9 years (e.g.,^18^). This improvement in memory representations of single events is attributed to hippocampal maturation at around 6 years^32,33^; nevertheless, memory binding may occur even before that age, but perhaps in a considerably less reliable or less strongly integrated fashion^31^. However, most studies on EM have focused on their maturation in older children in comparison to memory abilities in adults but not on their initial emergence in early childhood, making it unclear what is the exact developmental trajectory of memory-binding process in EM. This may be partly due to the limitations of the commonly used EM paradigms which often rely on designs that make them challenging to use with younger or children with atypical populations (computer-based or verbal tasks, e.g.^34^). Moreover, most nonverbal EM studies have utilized designs involving objects and locations on a computer screen^18,35^ and only few of them have tested EM development in first-person 3D space (e.g.^21,36,37^). To overcome the limitations of past studies, we created a simple nonverbal task, involving actual physical movement of the participant in real space with real objects (rather than just observation) to induce true EM of personal experiences (see Figure1 and Method section for a detailed description).

## Results

### Age-related differences in binding components across TD

**Figure 2:**
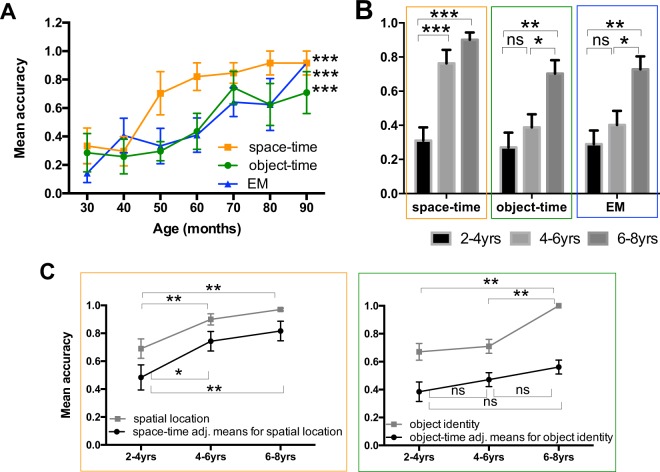
Early emergence of space-time binding across TD. (**A)** The graph presents the proportion of accurate responses across 2 to 8 years of age for *space-time*, *object-time*, *EM*. Space-time binding emerges first, followed by the other two conditions. Significant correlations were found between age as continuous variables and the three measures. (**B)** The graphs present the accuracy means for Space-Time, Object-Time and EM Tests in the TD sample divided by age-groups (2–4; 4–6; 6–8 yrs). (**C)** The graphs present the means for spatial location accuracy, and space-time binding adjusted for spatial location accuracy (left), and for object identity accuracy, and object-time binding adjusted for object identity accuracy (right). Adjusted means were calculated using GZLM. ns; p < 0.05 *p < 0.01 **p < 0.001 ***.

Figure 2A shows performance in the three tests across development. With age in months as a continuous variable, a Spearman’s correlation between age and each memory-binding component was performed. We found a strong correlation between the variables (*age* and *space-time*: r = 0.536, p < 0.001; *age* and *object-time*: r = 0.417, p < 0.001; *age* and *full EM*: r = 0.459, p = <0.001). To assess developmental change, we split the subjects into three age groups, 2–4, 4–6, and 6–8 years old. First, we calculated effect sizes (using the average of the three tests), comparing two groups at a time: 2–4 *vs.* 4–6-year-olds (large *Cohen’s d = *0.93); 4–6 *vs.* 6–8-year-olds (large *Cohen’s d = *1.14); 2–4 *vs.* 6–8-year-olds (large *Cohen’s d = *2.32; see descriptive statistics in Supplementary Information).

Performance on all three tasks varied significantly by age-group (Kruskal Wallis one-way ANOVA (Fig. 2B) H_(2)_ = 12.278, p < 0.01; H_(2)_ = 23.575, p < 0.001; H_(2)_ = 12.178, p < 0.01, respectively). 2–4-year-olds and 4–6-year-olds differed significantly only in Space-Time Test (H_(2)_ = −20.042, p = 0.001, Bonferroni-corrected). In the other two tests, 2–4- and 4–6-year-olds performed equally well (Object-Time Test: (H(2) = −5.565, p = 1.000; EM Test: H(2) = −4.208, p = 1.000) and were both significantly worse compared to 6–8-year-olds children (Object-Time Test: 2–4 year-olds, H_(2)_ = −18.934, p < 0.01; 4–6-year-olds, H_(2)_ = −13.278, p < 0.05; EM Test: 2–4-year-olds, H_(2)_ = −18.282, p < 0.01; 4–6-year-olds, H_(2)_ = −14.074, p < 0.05). These results suggest an early maturation of space-time binding between 2–4 years of age, followed by a protracted development of object-time binding and EM. In order to ensure that these patterns of results cannot be attributed simply to a memory of motor movements, we tested a subset of subjects in a “gated condition” during which a plastic gate was introduced right in front of the child between the encoding and retrieval phases. In order to perform the task, the child was required to walk around the gate forcing him to take a roundabout path that was different from the encoding phase (see Method section). We found no differences in performance between the gated and default conditions (Space-Time Test U = 9.500 p = 0.548; Object-Time Test U = 5.500 p = 0.151; EM U = 8.000 p = 0.421, Mann-Whitney U Test).

In the default condition, when looking just at children’s choice of locations and objects, without taking their temporal sequence into consideration, children performed well over chance across all ages (locations: 2–4-year-olds: 0.69 ± 0.08; 4–6-year-olds: 0.90 ± 0.04; 6–8-year-olds: 0.97 ± 0.02 paired two-tailed t-test p < 0.001, p < 0.001, p < 0.001, respectively; objects: 2–4-year-olds: 0.67 ± 0.06; 4–6-year-olds: 0.70 ± 0.05; 6–8-year-olds: 1 ± 0.00 paired two-tailed t-test p < 0.001, p < 0.001, p < 0.001, respectively) indicating that young children do not have difficulty recognizing the objects and places themselves (Fig. 2C, gray lines). Nevertheless, there is significant improvement for both over development (Kruskal Wallis one-way ANOVA, H_(2)_ = 16.835, p < 0.0011; H_(2)_ = 28.696, p < 0.001, respectively), with 2–4-year-olds performing worse than both 4–6- and 6–8-year-olds in remembering spatial locations (H_(2)_ = −14.229, p < 0.01, H_(2)_ = −18.991, p < 0.001, respectively, Bonferroni-corrected), and 6–8-year-olds performing better than both 2–4- and 4–6-year-olds in object recognition (H_(2)_ = −24.750, p = 0.0001, H_(2)_ = −21.188, p < 0.001, respectively, Bonferroni-corrected).

In order to address the possibility that early development of space-time binding could simply be an artifact of improvement in children’s spatial memory (and not in its temporal organization), a Generalized Linear Model (GZLM) was performed using space-time binding accuracy as the dependent variable, age-groups as the independent factor and spatial location accuracy as a covariate (Fig. 2C black line, left). Performance in the Space-Time Test still varied significantly between the three age-groups (F_(2) = _6.755, p < 0.01). 2–4-year-olds (0.48 ± 0.07) differed significantly from 4–6- and 6–8-year-olds in space-time binding, even when adjusted for spatial location accuracy (0.74 ± 0.05, p < 0.05; 0.82 ± 0.05, p < 0.01, respectively, Bonferroni-corrected), suggesting that the variance in the model is not explained simply by spatial memory, but by the developmental changes in the binding of space and time. Similar analyses were performed on the Object-Time Test (Fig. 2C black line, right), using object-time binding accuracy as the dependent variable, age-groups as the independent factor and object identity accuracy as a covariate. In contrast with the Space-Time Test, the Object-Time Test showed no significant differences across age-groups once it was adjusted for object choice accuracy (F_(2)_ = 0.871, p = 0.423), suggesting that the age effect in object-time binding is mostly driven by improvements in object recognition. Altogether, these results suggest that the development of temporally ordered representation of places does not simply rely on spatial memory but on the specific development in space-time binding (and that this developmental pattern is not true for object-time binding).

Additionally, we wanted to address the possibility that the difference among age-groups could simply stem from age-related differences in working memory (and not long-term hippocampus-based memory). Because we had conducted the Corsi Block-Tapping Task in each subject, we used children’s performance in that task as a measure of visuospatial working memory^38^ and conducted a GZLMusing space-time binding accuracy as the dependent variable and age-group as the independent factor, with Corsi performance as a covariate. Similar analyses were conducted with Object-Time and the full EM Test. The age effect remained significant in all three tests (F_(2)_ = 5.290, p = 0.009; F_(2)_ = 4.981, p = 0.011; F_(2)_ = 4.045, p = 0.025, respectively) even after accounting for age differences in WM, suggesting that the variance in the model is not explained by improvements in visuospatial short-term memory but by the developmental changes in long-term EM.

### Effects of space-time binding in EM representation

**Figure 3:**
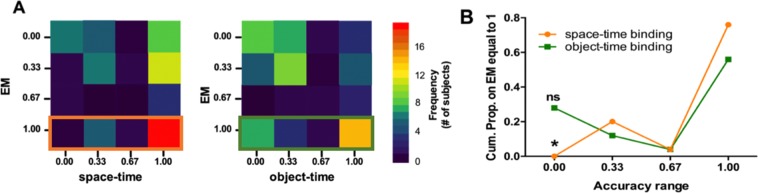
The role of space-time binding in EM. (**A)** Figures show the frequencies of the co-occurrences between each EM Test score and accuracy on the Space-Time Test (left), and Object-Time Test(right). (**B)** Presents the respective cumulative proportions of the Space-Time Test and Object-Time Test on accurate scores (equal to 1) on the EM Test. On the basis of the ordinal regression, when accuracy on the Space-Time Test is equal to 0 (failure in binding space and time), the probability of scoring high on the EM Test is significantly lower. ns = not significant; *indicates p < 0.05.

In order to investigate whether space-time binding ability might play a role in EM development, we conducted an ordinal regression analysis using performance in the Space-Time Test and Object-Time Test as predictors and performance in the EM Test as the dependent variable, with age as a covariate (see Fig. 3A,B). All the variables, with the exception of age, had an ordinal distribution representing four scores ranging from 0 (“Not Accurate”) to 1 (“Strongly Accurate”). The model was statistically significant (χ^2^_(7_) = 24.417, *p* = 0.001), showing a significant relationship between EM (scored 1, as the referent value) and total failure in the Space-Time Test (Wald χ^2^_(1)_ = 4.067, p < 0.05; *negative logit regression*: Exp (B) = 0.137) (the Wald test evaluates whether it is likely that the estimated effect could be zero). This result means that the failure in the Space-Time Test reduces the probability of being strongly accurate in the EM Test. In other words, successful EM rarely occurs without successful space-time binding, but this is not true for object-time binding. These results are in line with past studies^7,8^ emphasizing the role of space-time binding as a scaffold for episodic memories and shed light on the developmental emergence of this cognitive ability.

### Detailed analysis on the EM Test components: The binding of objects to space-time. 

**Figure 4:**
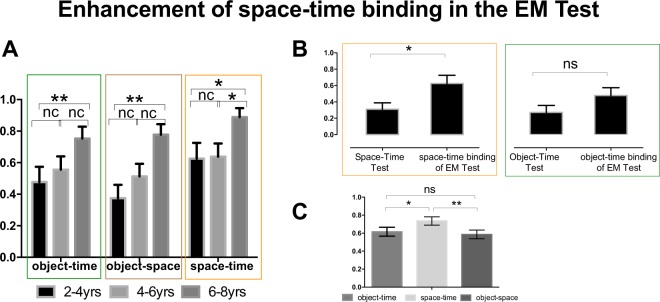
Enhancement of space-time binding in the EM Test. (**A)** The graphs present the accuracy means for object-time, object-space, and space-time binding components of the EM Test in the TD sample divided by age-groups (2–4; 4–6; 6–8 yrs). (**B)** Presents the accuracy means of Space-Time Test compared to space-time binding scores of the EM Test (left), and Object-Time Test compared to object-time binding of the EM Test for 2–4-years old children (right). (**C)** Presents the distribution of each EM binding components across all TD subjects. ns; p < 0.05 *p < 0.01 **.

We performed a detailed analysis on the EM Test components (i.e. spatial location, object identity, space-time binding, object-time binding, and object-space binding) in order to investigate whether developmental changes in space-time binding can be observed even within the EM Test itself. Figure 4A shows the distribution of each

EM Test binding component in children, divided by age-groups. Performance on space-time and object-space binding varied significantly by age-group, while object-time did not show any significant differences (Kruskal Wallis one-way ANOVA, *object-time:* H_(2)_ = 5.437, p = 0.066*; space-time*: H_(2)_ = 8.678,; p < 0.05 *object-space*: H_(2)_ = 12.029, p < 0.01). 2–4-year-olds and 6–8-year-olds differed significantly in space-time and object-space (H_(2)_ = −13.133, p < 0.05; H_(2)_ = −19.075, p < 0.01, respectively), while 4–6-year-olds and 6–8-year-olds differed significantly only in space-time (H_(2)_ = −11.769, p < 0.05). Interestingly, 2**–**4-year-olds perform as well as 4–6-year-olds children in their ability to bind space and time components in the EM Test. Surprisingly, if we compare these results with those obtained in the Space-Time Test (Fig. 4B, left), we observe that 2–4-year-olds do *better* in the space-time component of the EM Test (choosing the correct location in the correct order, regardless of object identity) than the Space-Time Test, in which object identity was held constant (Z = −2.137, p < 0.05, Wilcoxon signed-ranks test). In contrast, there is no significant difference between the object-time component of the EM Test (choosing the correct objects in the correct order, regardless of their location) in comparison with the Object-Time Test, in which spatial location was held constant (Z = −1.742, p = 0.082, Wilcoxon signed-ranks test); Fig. 4B, right).

How are even the youngest children able to bind space and time in the EM Test when the task requires a higher cognitive load, compared to the Space-Time Test? One possibility may be that because the EM Test was always conducted last, there is a practice effect. However, it is noted that such a practice effect is not found for object-time component. An alternative explanation is that the presence of objects strengthens spatiotemporal memory through the association of object content to spatial location. In fact, that may be the very mechanism by which objects get bound onto the spatiotemporal continuum of episodic memory. In Fig. 4C, it is possible to see that object-space binding follows a developmental pattern that highly resembles that of object-time. Consistent with this, space-time accuracy is significantly higher than both object-time and object-space accuracy, while object-time and object-space are not different (Z = 2.058, p < 0.05; Z = 2.795, p < 0.01; Z = −0.715, p = 0.475, respectively, Wilcoxon signed-ranks test).

When we conducted Spearman’s correlations between the binding components of the EM Test, we saw that, while these variables were strongly all related to one another according to zero-level correlations (object-time and object-space: r = 0.636, p < 0.001; object-time vs space-time: r = 0.361, p < 0.01; object-space vs space-time: r = 0.467, p < 0.001; Table 1), their partial correlations show a different result (see Table 2). In particular, if we partial out the effects of object-space binding, then object-time and space-time binding are no longer significantly correlated (r = 0.093, p = 0.456), suggesting that the successful binding of objects with time is mediated by the successful association between objects and its spatial context. In contrast, the other correlations remain largely unchanged. In other words, in the development of EM, the association of “what happened” with its spatial context may bring with it the temporal context (which has been already bounded to space) for free.

**Table 1:** Correlations between EM Test components.

	object-time binding	space-time binding	object-space binding	EM
object-time binding	1	0.361**	0.636**	0.819**
space-time binding	0.361**	1	0.467**	0.674**
object-space binding	0.636**	0.467**	1	0.859**
EM	0.819**	0.674**	0.859**	1

Pearson’s zero-order correlation for object-time, object-space, and space-time binding components and the total score of the EM Test in the TD sample.

**Table 2:** Partial correlations between EM Test components.

	Controlled for object-space binding		Controlled for object-time binding		Controlled for space-time binding	
	object-space binding	object-time binding	object-space binding	object-time binding	object-space binding	object-time binding
object-space binding	1	**0.093 (ns)**	1	0.330**	1	566**
object-time binding	**,093 (ns)**	1	0.330**	1	566**	1

Partial correlations between object-time and object-space binding components controlled for the effect of object-space, object-time and space-time binding. ns; p < 0.05 *; p < 0.01 **.

### Study 2: Atypical development of hippocampal binding components in Williams Syndrome

Williams Syndrome is a rare genetic disorder caused by a microdeletion of about 25 genes in region q11.23 of chromosome 7. WS patients are a particularly interesting population for investigating EM because of their abnormal hippocampal development and their unique cognitive profile. Individuals with WS show relative strengths in domains such as language production and face recognition but exhibit severe deficits in a range of spatial functions^19–22^. Pervasive impairment in the ability to construct arrays of objects with specific spatial relations among them^39–42^ is one of the most characteristic features of WS. Consistent with their visuospatial deficits, WS individuals are impaired in short^43,44^ and long-term^45^ tests of visuospatial memory. Importantly, however, their impaired long-term visuospatial performance is not merely due to difficulties in spatial working memory: WS show a greater memory decay effect between short and long delays, suggesting a specific deficit of consolidation of spatial memory. Such memory deficits documented in WS extend into their allocentric spatial navigation abilities^46^, as well as their associative memory for pairs of items^47^ and word-location pairs^48^. While there is a large body of literature on spatial memory in WS, no study has specifically investigated the ability to represent temporally continuous memories in this population. Therefore, Study 2 tested WS subjects (compared to TD controls) in the same task described in Study 1, in order to selectively assess the role of single EM components and their binding with time, as well as to speculate about the effects of atypical hippocampal development.

## Results

### Impairment in space-time binding and its impact on EM in WS

**Figure 5:**
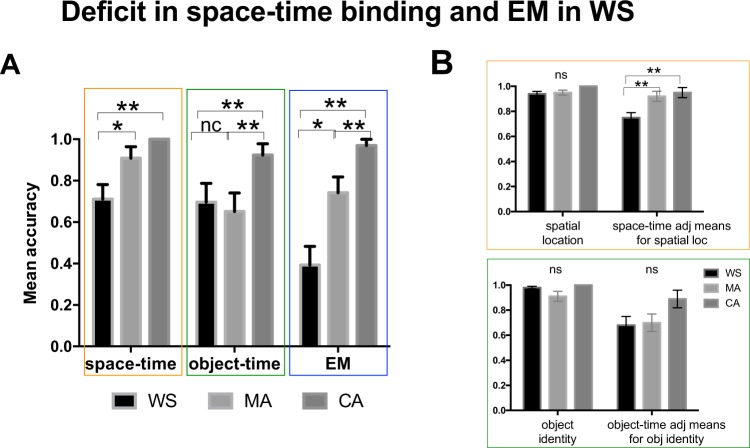
Deficit in space-time binding and full EM in Williams Syndrome. **(A)** The graphs represent the accuracy means for Space-Time, Object-Time and EM tests in WS patients, compared to MA and CA controls. (**B)** The graphs present the mean accuracies for spatial location and space-time binding adjusted for spatial location performance (above), and for object identity and object-time binding adjusted for object identity accuracy (below). Adjusted means were calculated using GZLM. ns = not significant. p < 0.05 *, p < 0.01 **.

We investigated memory binding processes in WS subjects, by comparing their performance with those of mental age-matched (MA) and chronological age-matched (CA) control subjects (Fig. 5A).

We first calculated effect sizes using the average of the three tests, comparing two groups at a time: WS *vs.* MA (large *Cohen’s d* = 0.68); WS *vs* CA (large *Cohen’s d* = 1.67); MA *vs.* CA (large *Cohen’s d* = 1.15; see descriptive statistics in Supplementary Information). Performance on the three tests significantly differed across groups (Independent-samples Kruskal Wallis one-way ANOVA: Object-Time Test: H_(2)_ = 6.823, p < 0.05; Space-Time Test: H_(2)_ = 14.658, p = 0.001; EM Test: H_(2)_ = 23.004, p < 0.001). WS participants performed significantly worse compared to MA controls in space-time binding and in EM (H_(2)_ = −10.409, p < 0.05; H_(2)_ = −13.568, p < 0.05, respectively, Bonferroni-corrected), while their memory for ordered recall of items was only delayed compared to CA (H_(2)_ = −11.591, p < 0.05; Fig. 5A). Interestingly, spatial location and object identity accuracy were both highly accurate and did not show any significant difference across the three groups (Fig. 5B).

In order to address the possibility that the failure in space-time binding could be driven simply by a deficit in WS subjects’ spatial memory and not its relation to temporal continuity, we performed a GZLM with accuracy in the Space-Time Test as the dependent variable, groups (WS, MA, CA) as the independent factor and spatial location accuracy as a covariate (Fig. 5B above). Performance in the Space-Time Test differed significantly by group, having adjusted for spatial accuracy (F_(2) = _6.927, p < 0.001), with WS subjects (mean 0.75 ± 0.04) performing significantly worse than MA and CA controls (means and t-tests against WS: 0.92 ± 0.04, p = 0.01; 0.95 ± 0.04, p < 0.01, respectively, Bonferroni-corrected). These results suggest that the difference across groups is not solely driven by differences in spatial accuracy but by those in space-time binding. A similar analysis was conducted using performance in the Object-Time Test as a dependent variable, groups as an independent factor and object identity accuracy as a covariate (Fig. 5B below). We found that the difference in object-time binding across groups was no longer significant once it has been adjusted for object accuracy (F_(2) = _2.299, p = 0.109). All together, these results suggest that the deficit in space-time memory in WS subjects does not simply rely on spatial memory difficulties but on binding deficits. As was the case for typically developing children in Study 1 (see Fig. 2C), temporal information was particularly relevant for explaining group differences when the task required recalling a sequence of locations rather than objects.

In order to investigate whether WS subjects’ performance can be explained by general cognitive ability, we conducted Spearman’s rank order correlations between WS subjects’ mental age, performance in Space-Time and Object-Time tests, and spatial location and object identity recognition accuracy. A significant correlation was found only between mental age and spatial location accuracy (r = 0.54, p < 0.01) but not between mental age and space-time binding (r = 0.24, p = 0.355). This means that as mental age (and general intelligence) increases in WS, location (but not temporal) memory improved; in other words, space-time binding deficits in WS patients is specific and is not related to their general cognitive abilities.

## Discussion

In Study 1, we set out to investigate whether space-time binding provides a scaffold upon which EM is constructed over development. First, we found that the ability to fully bind the *what*, *when*, and *where* components of an event develops around 6 years of age, consistent with other studies of episodic memory in children e.g.,^49^. Additionally, we showed here for the first time that the emergence of EM is preceded by the development of a competence in coherently binding together spatial and temporal information from 2 to 4 years of age and that this particular memory-binding component has a strong relationship with EM ability across individuals. Moreover, children’s ability to embed objects with time is significantly correlated with their ability to bind those objects to space, which may indicate that the binding of *what happened* with a spatiotemporal framework is necessary for successful EM.

The interpretation that the association of object content to spatial context is what allows objects to be embedded in EM is supported by direct recordings of spatially-selective neuronal activity in the hippocampus of both human and non-human animals during object memory recall^7,50^ and is consistent with theories that posit that the spatiotemporal context functions as a primary scaffold for memories of events^7,8^. However, our results add to that view behavioral and neuropsychological evidence on the origins of the basis for such EM representations in early human development. Our findings are also consistent with previous studies on cognitive development indicating a significant improvement in the use of multiple cues (e.g., environmental boundaries and landmarks) in spatial navigation around 5 or 6 years of age^51^; such changes in cue-binding abilities could be related to the maturation of EM in early childhood. Researchers suggest^52^ that the development of two shared mechanisms underlies both episodic memory and allocentric navigation: pattern separation, necessary to distinguish different memories and supported by the dentate gyrus^53^, and pattern completion, needed to integrate the elements of a memory and supported by CA3 and CA1^54^. While the early binding of space with time could be supported by temporal representations which have been found both in the hippocampus and in the entorhinal cortex [e.g.,^15^], the protracted maturation of the dentate gyrus^55^ as well as its connections with CA3^56^ might underlie the late emergence of fully bounded EM.

In Study 2, we investigated how the emergence of memory-binding abilities is affected by a deficit in spatial cognition accompanied by abnormal hippocampal development in WS. We showed that WS participants, unlike 4-year-old TD children, struggle with space-time binding and perform even worse when attempting to bind object contents inthe full EM Test.

Importantly, our results showed that WS was not impaired on their recognition of spatial location, indicating that their memory binding deficit is not driven solely by their spatial cognitive impairment. In fact, children and WS subjects were highly accurate in picking out the correct boxes and objects; they just could not embed them into a continuous episode. This finding is consistent with past work that shows a dissociation between recognition processes and memory-binding in TD children^18,34,49^ and a preserved object recognition ability in WS^47^. Accurate memory for the spatial context of objects has been linked to activity in the anterior hippocampus^33,57^, while the posterior hippocampus is correlated with navigation expertise^58^. Given that the hippocampus of WS individuals exhibits structural and functional abnormalities along the anterior-posterior axis, we might speculate that their impairment in spatiotemporal binding may be related to their smaller posterior hippocampus and that their difficulty in further binding elements of memory into a spatiotemporal context may be related to the decreased activity in the anterior hippocampus.

Interestingly, despite a clear and consistent deficit in spatiotemporal and episodic recall, the spared memory for sequences of objects in WS suggests an alternative strategy of object-related processing that might rely, in part, on connections with the prefrontal cortex^59^. This object-related success is consistent with spatial navigation studies that show better performance^46^ and qualitatively different behavior^60^ in the use of landmarks in WS compared with TD children.

## Conclusions

Time seems to have a special relation to space in that the first component of episode-like memory to emerge in development and to be impaired in WS is the binding of spatial and temporal information. Not only that, time seems to depend on its binding with space in order to weave together conceptual content, such as object identity, into its folds. These findings raise the possibility that the neural circuitry underlying episodic memory, in which the HF acts as a hub of information^32,61^, prioritizes spatiotemporal binding, which is ultimately what makes mental time travel possible. Further progress in our understanding of how EM develops in children and how they become impaired with hippocampal dysfunction is crucial for several reasons. First, the neural mechanisms underlying EM has been implicated not only in memory but in imagination or future planning^62^. Understanding how EM abilities can change over the course of development or neurological disorders will indirectly shed light on the emergence (and impairment) of high-level cognitive abilities such as creativity and imagination. Second, it has been proposed that the hippocampus adaptively organizes episodic memories into conceptual knowledge that drives adaptive novel behavior^63^. Insight into the origins of hippocampus-dependent memory binding mechanisms may help characterize how abstract conceptual maps can form and be modified in human cognition.

## Methods

### Participants

#### Study 1

67 children (34 females) between the ages of 2.52 and 8.74 (M = 5.53; SD = 1.63) participated in Study 1. Children were recruited through fliers delivered to daycares, preschools and elementary schools. Once parents agreed to have their child participate by signing up on the lab’s online form, they were contacted for an appointment. All children were tested at the Developmental Cognitive Neuroscience Lab at the Center for Mind/Brain Sciences, University of Trento. Children received a small gift for their participation. Most children came from Rovereto and its surrounding areas. 11 additional children from 2 to 4 years of age were excluded from the study due to medical problems, extreme prematurity, important language development delays, failure to complete the task, or refusal to follow instructions.

#### Study 2

27 Williams Syndrome subjects participated in Study 2 and were matched with TD control participants with similar mental and chronological ages. Because the age range proved to be too heterogeneous, ranging from children as young as 6 year old to 40-year-old adults, we decided to exclude all child participants (under the age of 12) in order to create a more homogeneous sample. Therefore, the final sample considered for analysis consisted of 22 WS subjects (11 females), 22 mental-age-matched controls (11 females) and 22 chronological-age-matched controls (11 females).

The WS participants belonged to two different Italian Williams Syndrome associations: AGSW (Associazione Genitori Sindrome di Williams) and AFSW (Associazione Famiglie Sindrome di Williams). Families were contacted by telephone or email after parents had agreed to have their child participate in the study. Subjects were tested in three different locations: during two different yearly WS retreats in Fano and Peschiera and by appointment at the Clinical Genetic Unit, Fondazione IRCCS Ca Granda Ospedale Maggiore Policlinico in Milan. TD control children and adults were tested at the Developmental Cognitive Neuroscience Lab of the Center for Mind/Brain Sciences, University of Trento. They were recruited either from Rovereto and surrounding areas or by contacting families of children who had previously participated in studies at the lab. Importantly, in each location we recreated the same experimental environment in terms of room size and landmark positions. Each WS participant was individually matched for mental and chronological age, gender and trial order with TDcontrols. WS subjects ranged in chronological age from 12.86 to 43.78 (M = 24.84; SD = 8.11) and mental age range from 5.11to 8.96 (M = 6.44; SD = 0.98). For a measure of non-verbal mental age, WS participants were administered four subscales of the Brief IQ Visualization and Reasoning tests from the Leiter International Performance Scale Revised (Leiter-R).

### Procedure

Participants were tested on a non-verbal object-placement task developed to allow isolated testing of the *what*, *where*, and *when* components that make up an episodic memory. Each experimental session was composed of three tests (Fig. 1): The Space-Time Test was designed to measure the ability to remember spatial locations and organize them in time but did not require object recognition. The Object-Time Test measured the ability to remember different objects in a temporal order, while keeping spatial location constant. Finally, the EM Test assessed the ability to combine object identity, spatial locations, and their temporal order together into one representation. All subjects completed the three tests. The Space-Time Test and Object-Time Test were counterbalanced in their order, and then the EM Test was always presented last. The selected objects, locations, and temporal sequences were always randomized across subjects and within trials. The task was straightforward: the experimenter sequentially placed three objects in boxes laid out in the experimental room while the participant was watching (demonstration phase), then the participant was asked to do the same thing by placing the objects himself/herself (encoding phase). After a delay during which a verbal interference task was administered, the participant was asked to re-enact his own previous actions (retrieval phase).

Before beginning the experiment, two training tasks were provided in order to elicit imitation behaviors in children. In the first one, the experimenter hid one of three colored crayons in one of three grey plastic cups, while in the second one the experimenter demonstrated some motor sequences while standing up and facing the participant. The participant was then asked to repeat the experimenter’s behavior. If the participant struggled to understand or failed to reproduce the correct behavior, the experimenter repeated the sequence while emphasizing the relevant information. The training ended after the participant had successfully copied the experimenter twice.

To start the experiment, the experimenter brought the participant to the starting position located at one end of a rectangular room, in front of a large door. Once the participant was standing in front of the door and facing the room, the experimenter proceeded to give the instructions verbally by using an animal finger puppet (e.g., a lion) and telling the following short story: “You know, this little lion loves cookies; she always bakes too many and then has to hide them. Now, pay attention carefully to how I hide them, because afterwards you will have to do it just like me. Otherwise the lion will not be able to find them later!” The participant was then shown a plastic tray with the “cookies” (objects) that the animal had “baked” and was given about five seconds to observe them or manipulate them. The tray was then placed on an adjacent shelf, out of view from the child. Then the participant watched the experimenter hide three objects into a subset of the five boxes that were distributed across the room. For the Space-Time Test, children were provided with three identical objects that they had to place into three of the five boxes in the room. For the Object-Time Test, children had to choose three of five different objects to place into a single box nearby. For the EM Test, children had to choose three of five different objects, and place them into three of five different boxes. The objects were always hidden one at the time: the experimenter would pick up one, show it to the child for a couple seconds, before proceeding to hide it, and then return to the starting point. After each of the three objects were placed into a box, the experimenter led the child to face away from the boxes under the pretense of hiding from strong winds, while another experimenter collected the objects and returned them to the tray.

For the participant to encode his/her own episodic experience, the encoding phase involved the participant being presented with the same objects in the tray and instructed to “hide the cookies” just like the experimenter did. If the participant attempted to pick up more than one object at once, the experimenter would remind him/her to hide them one at the time. Once each of the three objects had been hidden, child and experimenter would again face away while they were picked up by a second experimenter. After the encoding episode, an interference task was administered. The participant and the experimenter sat down together at a table present in the room under the pretense of teaching the animal puppet a few words. A verbal naming task was performed for a total of 3 minutes. Finally, during the retrieval phase, the participant and experimenter returned to their starting position by the door, and the participant was presented with the objects and asked to “hide the cookies” as she/he had previously done. After each trial, the participant was rewarded with a sticker (regardless of his/her performance).

In order to investigate the potential effect of learning a particular trajectory or motor sequence, 5 subjects were tested in a “gated condition” during which a plastic gate was introduced right in front of the child between the encoding and retrieval phases. In order to perform the task, the child was required to walk around the gate, through a different route than the one previously taken during the encoding phase. There were no differences children’s memory performance in this condition, as compared to the rest (Space-Time Test *U* = 9.500 p = 0.548; Object-Time Test *U* = 5.500 p = 0.151; EM *U* = 8.000 p = 0.421, Mann-Whitney U Test).

### Scoring

By comparing the subject’s accuracy between the encoding and retrieval phases, we calculated the accuracy in the participant’s memory for the single components (object identity or spatial location) and the extent to which they were able to bind them in a temporal sequence (object-time binding; space-time binding; EM). For the individual element analysis, the space location and object identity accuracy were calculated by giving a point for each correct remembered location or object, respectively. For temporal binding, three scores were calculated: space-time binding, for each correctly-remembered location in the correct place in the sequence; object-time binding, for each correctly-remembered object in its correct temporal sequence; EM, giving a point for each different object that is correctly remembered in its correct spatial *and* temporal position (general EM score). The general EM score was decomposed into three further component scores representing space-time, object-time, and object-space binding. For simplicity, each index was calculated on the basis of a proportion of correct scores (maximum raw score being 3). See the Supplementary Information for a detailed description of procedures and scoring system.

This study was conducted in accordance with the guidelines of the Human Research Ethics Committee of the University of Trento (internal review board approval No. 2016-005). The experimental protocol was approved by the internal review board, and informed consent was obtained from both the participants and their parents or legal guardians.

## Supplementary information

Supplementary Information

## Data Availability

The datasets generated during and/or analysed during the current study are available from the corresponding author on reasonable request.
