# Author Correction: The spatiotemporal organization of episodic memory and its disruption in a neurodevelopmental disorder

**DOI:** 10.1038/s41598-020-75727-w

**Published:** 2020-10-28

**Authors:** Marilina Mastrogiuseppe, Natasha Bertelsen, Maria Francesca Bedeschi, Sang Ah Lee

**Affiliations:** 1 Center for Mind/Brain Sciences (CIMeC), University of Trento, Rovereto, Italy; 2 Department of Human Studies, University of Trieste, Trieste, Italy; 3 Center for Neuroscience and Cognitive Systems (CNCS), Italian Institute of Technology, Rovereto, TN Italy; 4 Medical Genetic Unit, Woman-Child-Newborn Department, Fondazione IRCCS Ca ‘Granda Ospedale Maggiore Policlinico, Milan, Italy; 5 Department of Bio and Brain Engineering, Korea Advanced Institute of Science and Technology, Daejeon, Korea

Correction to: *Scientific Reports* https://doi.org/10.1038/s41598-019-53823-w, published online 05 December 2019

This Article contains errors. In Figures 4A and 5A “ns” is incorrectly written as “nc”. The correct Figures 4 and 5 appear below as Figures 1 and 2.

**Figure 1:**
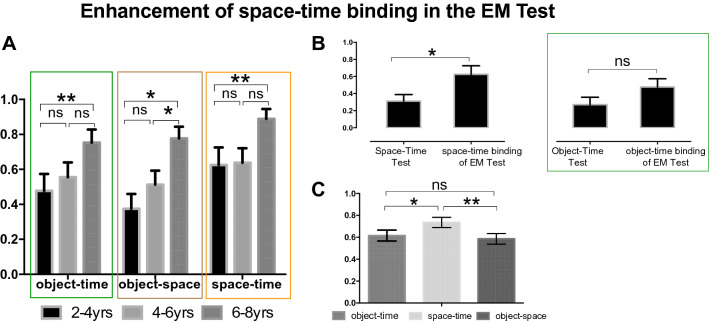
Enhancement of space-time binding in the EM Test. (**A**) The graphs present the accuracy means for object-time, object-space, and space-time binding components of the EM Test in the TD sample divided by age-groups (2–4; 4–6; 6–8 yrs). (**B**) Presents the accuracy means of Space-Time Test compared to space-time binding scores of the EM Test (left), and Object-Time Test compared to object-time binding of the EM Test for 2–4-years old children (right). (**C**) Presents the distribution of each EM binding components across all TD subjects. ns; p < 0.05 *p < 0.01 **.

**Figure 2:**
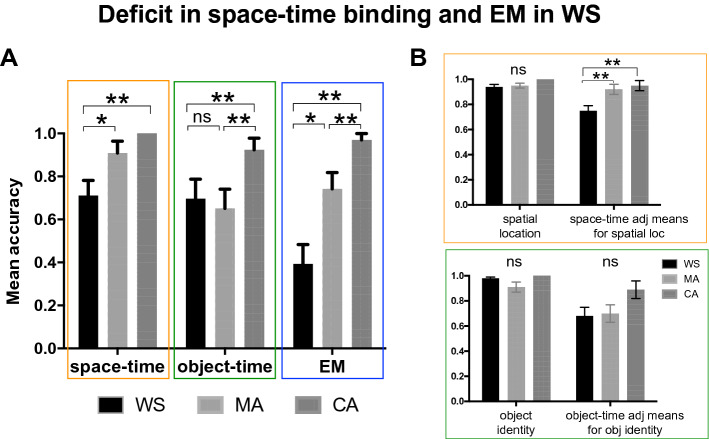
Deficit in space-time binding and full EM in Williams Syndrome. (**A**) The graphs represent the accuracy means for Space-Time, Object-Time and EM tests in WS patients, compared to MA and CA controls. (**B**) The graphs present the mean accuracies for spatial location and space-time binding adjusted for spatial location performance (above), and for object identity and object-time binding adjusted for object identity accuracy (below). Adjusted means were calculated using GZLM. ns = not significant. p < 0.05 *, p < 0.01 **.

In addition, the legend for Table 1 is incorrect.

“Correlations between EM Test components. Pearson’s zero-order correlation for object-time, object-space, and space-time binding components and the total score of the EM Test in the TD sample.”

should read:

“Correlations between EM Test components. *A.* Pearson’s correlation for object-time, object-space, and space-time binding components and the total score of the EM Test in the TD sample. *B.* Partial two-tails correlations between object-time and object-space binding components controlled for the effect of object-space, object-time and space-time binding. ns; p < 0.05 *; p < 0.01 **”

Finally, in Table 2, the value “.566**” is incorrectly given as “566**”, and the value “.093 (ns)” is incorrectly given as “,0.93 (ns)” and “0.093 (ns)”, and the value “.330**” is incorrectly given as “0.330**”.

The correct Table 2 appears below as Table 1.

**Table 1:** Partial correlations between EM Test components. Partial correlations between object-time and object-space binding components controlled for the effect of object-space, object-time and space-time binding. ns; p < 0.05 *; p < 0.01 **

	Controlled for object-space binding		Controlled for object-time binding		Controlled for space-time binding	
	Object-space binding	Object-time binding	Object-space binding	Object-time binding	Object-space binding	Object-time binding
Object-space binding	1	**.093 (ns)**	1	.330**	1	.566**
Object-time binding	**.093 (ns)**	1	.330**	1	.566**	1

