# Supplementary material for: The spatiotemporal organization of episodic memory and its disruption in a neurodevelopmental disorder
Source: Sci Rep. 2019 Dec 5;9:18447. doi: 10.1038/s41598-019-53823-w (PMC6895173; doi:10.1038/s41598-019-53823-w)
Supplement: Supplementary file 1 — Supplementary Information [file 41598_2019_53823_MOESM1_ESM.docx]

**Online Support Material for “The spatiotemporal organization of episodic memory and its disruption in a neurodevelopmental disorder”**

**Marilina Mastrogiuseppe^a^, Natasha Bertelsen ^a^, Maria Francesca Bedeschi ^b^, Sang Ah Lee^c*^**

^a^Center for Mind/Brain Sciences, University of Trento, Rovereto, TN, Italy

^b^ Clinical Genetic Unit, Fondazione IRCCS Ca’ Granda Ospedale Maggiore Policlinico, Milan, Italy

^c^Department of Bio and Brain Engineering, Korea Advanced Institute of Science and Technology, Daejeon, Korea

In this online support material, we provided a detailed description of the methods, including the materials, procedures (including the script translated from Italian), experimental sequences, and other instructions in order to facilitate future research using this task. We have also included a detailed description of the scoring system and mean raw scores for both Study 1 (Typical Development) and Study 2 (Williams Syndrome).

**Detailed Materials**

In total, thirteen objects were created by hand using clay, dried and painted. Of these, three were identical (e.g., 5-cm-long green sticks) and used in the Space-Time Test to exclude the object recognition component of episodic memory. The ten remaining objects were created in all different shapes and colors (See Image 1 below), but roughly of the same overall size. Five of those objects were used in the Object-Time Test and the other five were used in the EM Test.

For the location component of the experiment, 5 identical 20x30 cm cardboard boxes were distributed around the room in an asymmetrical configuration.


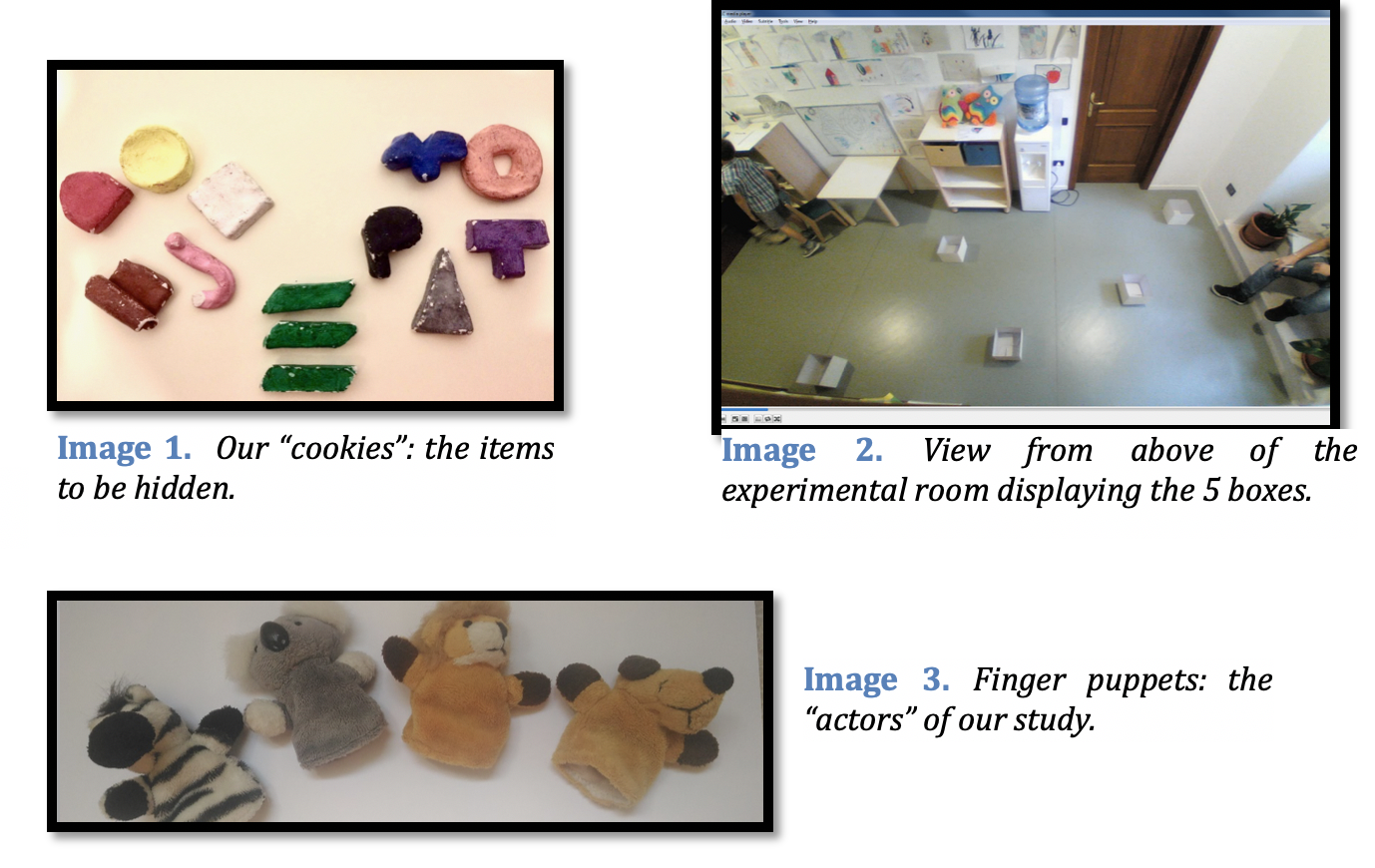


**Detailed Procedures**

Participants were welcomed into the lab (Developmental Cognitive Neuroscience Lab, Center for Mind/Brain Sciences, University of Trento, Rovereto), where toys and books were made available in order to familiarize the participant with the environment. The experimenter explained the study to the parent/guardian and answered any question they might have before obtaining their informed consent. After playing freely for a couple of minutes with the experimenter, the participant and the experimenter moved to the adjacent experimental room while the parent watched the live video-recording on the lab’s computer monitor from the next room.

Before beginning the experiment, two training tasks were administered. In the first one, the experimenter hid one of three colored crayons in one of three grey plastic cups placed in front of the participant. The participant was then asked to repeat the experimenter’s behavior. If the participant struggled to understand or failed to reproduce the correct behavior (for example by picking the wrong crayon and placing it in the wrong plastic cup) the experimenter repeated the sequence while emphasizing the choice of item and location. The training ended after the participant had successfully copied the experimenter twice. In the second training task, the participant was asked to copy a sequence of movement produced by the experimenter, while standing up and facing the participant. The first motor sequence was comprised of one action (e.g., *extend left arm above head*), a sequence of two actions (e.g., t*ake one step to the left and extend right arm to the right*), and a sequence of three actions (e.g., *jump in place, extend both arms up, then take a step backwards*).

After completion of the training trials, the experimenter brought the participant to the starting position, located by the door at one end of the room. Once the participant was standing in front of the door facing the array, the experimenter proceeded to give the verbally instructions for the task by showing the participant an animal finger puppet (e.g., a lion) and telling her/him the following short story: “*You know, this little lion is a glutton for cookies; she always bakes too many and we have to help her hide them. Now, pay attention carefully to how I hide them, because afterwards you will have to do it just like me. Otherwise she’s not going to find them!*” The participant was then shown a plastic tray with the specific cookies that the animal has “baked” and was given five seconds to observe them or manipulate them. The tray was then placed on a nearby shelf, out of view from the participant.

During the hiding phase, the participant stood at the starting point and watched the experimenter hide three objects into the boxes. The objects were always hidden one at the time: the experimenter picked up one, showed it to the participant for a couple seconds, then proceeded to hide it. After the three objects had been placed, the participant and the experimenter turned around to face away from the boxes (i.e., under the play pretense of hiding from strong winds) while another experimenter (“the wind”) brought back the objects to the tray.

During the encoding phase, the participant was presented with the object tray and instructed to “*hide the cookies*” just like the experimenter. If the participant attempted to pick up more than one object at once, the experimenter would remind him/her to hide them “*one at a time*”. Once three objects had been hidden, the participant and experimenter again faced away during their retrieval by “the wind”.

After encoding, a 3-minute interference task was administered. The participant and the experimenter sat down together at a small table present in the room under the pretense of teaching the animal puppet some words. The Peabody Picture Vocabulary Test (Italian) was then administered for a total of 3 minutes.

Finally, during the retrieval phase, the participant and the experimenter returned to their starting position by the door and the participant was presented with the object tray once again and asked to “*hide the cookies* *as s/he had previously done*.” After each retrieval phase, regardless of the accuracy of the task performance, the participant was rewarded with a sticker.

At the end of the three tests, the participant and the experimenter sat down at the table together, and the Corsi Block-Tapping test was administered. After completion, and if necessary, a few more minutes were spent on completing the Peabody test. At the end of the visit, the participant was given a small gift of their choice (e.g., cup, ball, toy) for participating in the study.

**Detailed Scoring**

The subjects’ performance was transcribed into a spreadsheet and scored: in each trial, points were given based on both single element recognition (correct object or location) and their ability to bind them (correct temporal order). As we were interested in long-term episodic memory and not in immediate imitation, we only compared the performance between encoding and retrieval, between which there was the interference task. In analyzing the data, we were mainly interested in the bound components of each trial: object-time (what+when), space-time (where+when) and full EM (what+where+when). However, we also calculated single indexes: object identity (what) for Object-Time Test, and spatial location (where) for Space-Time Test. This allowed us to have a reference of item and location recognition memory to compare to episodic memory binding. We also calculated Object-Time, Object-Space, and Space-Time binding components of the EM Test.

The details of scoring procedures are reported below:

In **Object-Time Test**, two different indices of performance were calculated:

- **index “object identity”**: *1 point for each correctly remembered object, maximum 3 points*.
- **Index “object-time” binding**: *1 point for each object that is correctly remembered in its absolute temporal position, maximum 3 points (e.g., 1^st^ = red; 2^nd^ = yellow; 3^rd^ = pink)*.

In **Space-Time Test**, the two different indexes were calculated:

- index **“spatial location”**: *1 point for each correctly remembered location, maximum 3 points*.
- index **“space-time” binding”**: *1 point for each object that is correctly remembered in its absolute spatial position, maximum 3 points (e.g., 1^st^ = box 4; 2^nd^ = box 2; 3^rd^ = box 5).*

In **EM Test**, we calculated the following index of performance:

- index **“full EM”**: *1 point for each object that was correctly remembered in its correct spatiotemporal position, maximum 3 points*.
- Index **“object-time” binding in EM:** *1 point for each object that was correctly remembered in its absolute temporal position, maximum 3 points.*
- Index **“object-space” binding in EM:** *1 point for each object that was correctly remembered in its absolute spatial position, maximum 3 points.*
- Index **“space-time” binding in EM:** *1 point for each spatial location that was correctly remembered in its absolute temporal position, maximum 3 points.*

**Descriptive Statistics**

| **Table 1**   \| Descriptive statistics of EM components by group in TD. \| \| --- \| | | | | | | | | | | | | | | | | |  |  |  |
| --- | --- | --- | --- | --- | --- | --- | --- | --- | --- | --- | --- | --- | --- | --- | --- | --- | --- | --- | --- | --- |
|  | | | | 2–4-year-olds | | |  | | | 4–6-year-olds | | |  | 6–8-year-olds | | | | | |
|  | | | | *M* | | *SD* | | |  | | *M* | *SD* |  | |  | *M* | | *SD* |  |
| **Space-Time Test** | | | |  | |  | | |  | |  |  |  | |  |  | |  |  |
| space-time binding | | | | .106 | | .30944 | | |  | | ,3106 | ,30944 |  | |  | ,9011 | | ,22365 |  |
| spatial location | | | | ,6881 | | ,31025 | | |  | | ,6881 | ,31025 |  | |  | ,9756 | | ,08807 |  |
| **Object-Time Test** | | | |  | |  | | |  | |  |  |  | |  |  | |  |  |
| object-time binding | | | | ,2700 | | ,34900 | | |  | | ,2700 | ,34900 |  | |  | ,7030 | | ,40720 |  |
| object accuracy | | | | ,6675 | | ,24466 | | |  | | ,6675 | ,24466 |  | |  | 1,0000 | | ,00000 |  |
| **EM Test** | | | |  | |  | | |  | |  |  |  | |  |  | |  |  |
| full EM | | | | ,2900 | | ,31891 | | |  | | ,2900 | ,31891 |  | |  | ,7281 | | ,39311 |  |
| object-time binding | | | | ,4775 | | ,38498 | | |  | | ,4775 | ,38498 |  | |  | ,7530 | | ,38813 |  |
| space-time binding | | | | ,6250 | | ,40195 | | |  | | ,6250 | ,40195 |  | |  | ,8889 | | ,29250 |  |
| object-space binding | | | | ,3744 | | ,34231 | | |  | | ,3744 | ,34231 |  | |  | ,7774 | | ,34666 |  |
|  |  |  |  | |  | | |  |  |  |  |  |  |  |  |  |  |  |  |
| **Table 2**   \| Descriptive statistics of EM components by group in WS and TD. \| \| --- \| | | | | | | | | | | | | | | | | |  |  |  |
|  | | | | WS patients | | |  | | | MA controls | | |  | CA controls | | | | | |
|  | | | | *M* | | *SD* | | |  | | *M* | *SD* |  | |  | *M* | | *SD* |  |
| **Space-Time Test** | | | |  | |  | | |  | |  |  |  | |  |  | |  |  |
| space-time binding | | | | ,7109 | | ,33169 | | |  | | ,9091 | ,25597 |  | |  | ,9241 | | ,25092 |  |
| spatial location | | | | ,9400 | | ,13027 | | |  | | ,9550 | ,11591 |  | |  | 1,0000 | | ,00000 |  |
| **Object-Time Test** | | | |  | |  | | |  | |  |  |  | |  |  | |  |  |
| object-time binding | | | | ,6964 | | ,42355 | | |  | | ,6509 | ,41818 |  | |  | ,9241 | | ,25092 |  |
| object accuracy | | | | ,9850 | | ,07036 | | |  | | ,9091 | ,21087 |  | |  | 1,0000 | | ,00000 |  |
| **EM Test** | | | |  | |  | | |  | |  |  |  | |  |  | |  |  |
| full EM | | | | ,3932 | | ,41982 | | |  | | ,7423 | ,35597 |  | |  | ,9695 | | ,14284 |  |
| object-time binding | | | | ,5595 | | ,40427 | | |  | | ,7732 | ,33170 |  | |  | ,9695 | | ,14284 |  |
| space-time binding | | | | ,5445 | | ,44346 | | |  | | ,8791 | ,30049 |  | |  | 1,0000 | | ,00000 |  |
| object-space binding | | | | ,5295 | | ,43277 | | |  | | ,8332 | ,26816 |  | |  | ,9695 | | ,14284 |  |
